# DNA Interaction with Head-to-Tail Associates of Cationic Surfactants Prevents Formation of Compact Particles

**DOI:** 10.3390/molecules23071576

**Published:** 2018-06-28

**Authors:** Nina Kasyanenko, Ivan Unksov, Vladimir Bakulev, Svetlana Santer

**Affiliations:** 1 Department of Physics, Saint Petersburg State University, 199034 St Petersburg, Russia; vanjaunksov@mail.ru (I.U.); vbakulev@inbox.ru (V.B.); 2 Experimental Physics, Institute of Physics and Astronomy, University of Potsdam, 14476 Potsdam-Golm, Germany; santer@uni-potsdam.de

**Keywords:** azobenzene trimethylammonium bromide, head-to-tail surfactant associates, DNA, ionic strength, multivalent ions

## Abstract

Cationic azobenzene-containing surfactants are capable of condensing DNA in solution with formation of nanosized particles that can be employed in gene delivery. The ratio of surfactant/DNA concentration and solution ionic strength determines the result of DNA-surfactant interaction: Complexes with a micelle-like surfactant associates on DNA, which induces DNA shrinkage, DNA precipitation or DNA condensation with the emergence of nanosized particles. UV and fluorescence spectroscopy, low gradient viscometry and flow birefringence methods were employed to investigate DNA-surfactant and surfactant-surfactant interaction at different NaCl concentrations, [NaCl]. It was observed that [NaCl] (or the Debye screening radius) determines the surfactant-surfactant interaction in solutions without DNA. Monomers, micelles and non-micellar associates of azobenzene-containing surfactants with head-to-tail orientation of molecules were distinguished due to the features of their absorption spectra. The novel data enabled us to conclude that exactly the type of associates (together with the concentration of components) determines the result of DNA-surfactant interaction. Predomination of head-to-tail associates at 0.01 M < [NaCl] < 0.5 M induces DNA aggregation and in some cases DNA precipitation. High NaCl concentration (higher than 0.8 M) prevents electrostatic attraction of surfactants to DNA phosphates for complex formation. DAPI dye luminescence in solutions with DNA-surfactant complexes shows that surfactant tails overlap the DNA minor groove. The addition of di- and trivalent metal ions before and after the surfactant binding to DNA indicate that the bound surfactant molecules are located on DNA in islets.

## 1. Introduction

Cationic surfactants can condense high molecular DNA in solutions to produce nanosized particles [1,2,3,4,5,6,7]. DNA condensation induced by a variety of agents is an important stage in gene delivery technique [8,9,10]. The compact structures are typically observed in the excess of cationic surfactant in DNA solution at a sufficiently high charge ratio z (the number of positive groups of surfactants per a single DNA phosphate) [4,5,7,11]. The amphiphilic nature of surfactants causes their cooperative binding to DNA [12]. Micelle-like surfactant associates on the DNA chain at concentrations below CMC (critical micelle concentration) can be observed at z < 1 in solutions of relatively low ionic strength [5,13,14]. They can induce DNA shrinkage due to thermodynamically preferred contacts of surfactant hydrophobic tails linked to distant sites on DNA [2,5]. DNA conformational transition (shrinkage of the DNA molecular coil at low [NaCl] at z < 1) should be distinguished from a phase transition with the formation of colloid-containing stable nanosized particles at z > 2 [5]. At low ionic strength, these conformational and phase transitions are separated by the range of z values at which DNA precipitates as loose flakes that does not occur at sufficiently low DNA concentration. Furthermore, the coexistence of DNA coils and globules in solutions have been discussed [4,15]. 

Photosensitive azobenzene-containing surfactants are of special interest. Their numerous technological and bio-related applications emerge due to the UV/Vis light-induced reversible trans-cis-trans isomerization. They can be employed as containers with photosensitive lock for delivery and release of drugs into cells [16], in biofilms, photoresponsive DNA thermotropic liquid crystals [17], for photoregulated antibacterial activity, and biological photocontrol [17,18,19,20]. The light-controlled reversible DNA packaging is well studied when using azobenzene-containing surfactants [1,5,11,21,22,23]. The length of the hydrophobic tail of surfactant influences the DNA packaging/unpackaging [3,24]. 

**Figure 1:**
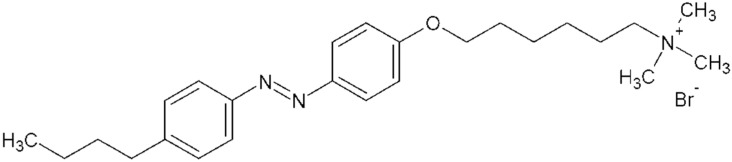
AzoTAB structure in trans-conformation.

In our research, the cationic azobenzene trimethylammonium bromide surfactant C_4_-Azo-OC_6_TMAB, AzoTAB (Figure 1) was used. It was observed that at low ionic strength the DNA shrinkage induced by AzoTAB is accompanied by the change in DNA persistence length [5,25]. The DNA persistence length does not decrease as a result of phosphates screening at [NaCl] > 0.005 M NaCl [26,27]. The significant change in DNA stiffness (persistence length), which is the critical conformation property of the DNA molecule clearly cannot be caused by electrostatic interaction of the surfactant with DNA. The drop in the persistence length can be induced by the local bending of the double helix at the site of surfactant binding.

The effect of NaCl concentration on the cationic surfactants interaction with DNA is of special significance despite extensive research in this field [12,14,28]. AzoTAB surfactant cannot cause DNA condensation at [NaCl] > 200 mM [24]. Moreover, this surfactant cannot induce visible DNA shrinkage in 1 M NaCl. It is known that CMC (critical micelle concentration) decreases with the growth of [NaCl]. The emergence of micelles in surfactant solution does not have an influence on DNA shrinkage at low NaCl concentrations (e.g., 0.005 M NaCl) or on phase transition in DNA-surfactant systems [4,5]. 

The first motivation of our work was to clarify the role of the salt concentration [NaCl] in DNA–surfactant interaction at z < 1. This is due to the fact that at [NaCl] from 0.01 M to 0.5 M it is not possible to prepare stable solutions of DNA with surfactants at sufficiently high DNA concentrations. In this regard, we first focused on the study of [NaCl] influence on the surfactant-surfactant interaction in solutions without DNA. Our second aim was to analyze the possibility of di- and trivalent metal ions binding to DNA after its complexation with surfactant. Such studies, as well as the analysis of the fluorescence of the DAPI dye in solutions with DNA-surfactant complexes, provide the information necessary for constructing an accurate model of surfactant binding to DNA

It is known that micelle formation depends on solution ionic strength for a number of reasons including entropy contribution, reduced repulsion and salt bridges between charged groups [2,29]. Non-micellar surfactant aggregates are less investigated than micelles. Large aggregates of azobenzene cationic surfactants were observed beyond CMC as vesicles [30]. H-aggregates and J-aggregates were also discussed for azobenzene surfactants [31]. The premicellar aggregation of different surfactants was discussed earlier [32,33]. For example, the parallel orientation of monomers with opposite location of their charged heads was proposed for dimers of anionic surfactants [34]. The formation of associates of cationic surfactants at sub-CMC concentrations was demonstrated by discrete changes in surfactant absorbance [35]. Thus, the major goal of our research was to elucidate the influence of ionic conditions on AzoTAB association, and consequently on AzoTAB–DNA interaction.

In order to characterize DNA-surfactant interaction, we analyzed the fluorescence of DAPI dye (4′,6-diamidino-2-phenylindole) in DNA-surfactant solution. The ability of DAPI to form complexes with DNA bases in the minor groove provides information about whether surfactants overlap access to these binding sites.

## 2. Results and Discussion

### 2.1. Influence of Salt Concentration on Surfactant-Surfactant Interaction in Solutions

**Figure 2:**
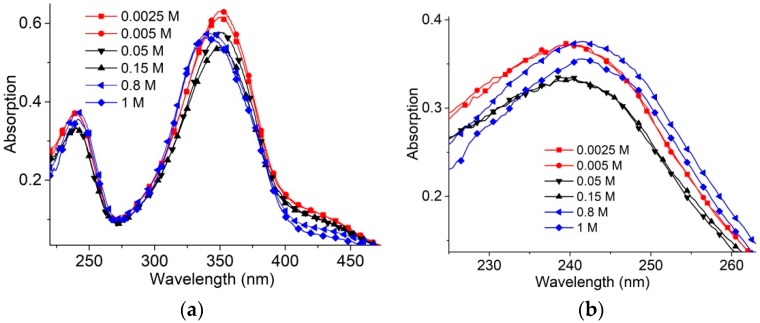
Surfactant absorbance spectra (**a**) at varied [NaCl] (as depicted) and zoomed-in short-wavelength band (**b**). [AzoTAB] = 8 × 10^−6^ M.

Electrostatic interactions essentially define the cationic surfactants binding to DNA. Long- and short-range electrostatic interactions determine DNA conformation (particularly polyelectrolyte swelling and persistence length). Polyelectrolyte swelling of DNA ceases at NaCl concentrations higher than 0.5 M, while the experimentally determined DNA persistence length remains unchanged at [NaCl] > 0.005 M. Salt concentration and surfactant concentration determine intermolecular interactions in surfactant solutions without DNA (Figure 2). In Figure 2a,b, three different types of surfactant absorption spectra are presented: at low (<0.01 M), high (>0.5 M) and intermediate [NaCl]. The hypsochromic (blue) shift of the absorption band at 300–390 nm and hypochromicity should be attributed to the AzoTAB micelles [5,30]. The changes in the band at 220–270 nm (Figure 2b) and at “shoulder” at wavelengths λ > 390 nm also support the formation of micelles at 0.8 M and 1 M NaCl. Surfactant absorption spectra at intermediate NaCl concentration demonstrate hypochromicity only (without shift). The band at 220–270 nm is due to the absorption of benzene rings. It has two clearly distinguished intensity levels for low and intermediate salt concentration before the emergence of the micelles at high [NaCl]. We can assume the appearance of associates with an orientation different from that realized in the micelles. The existence of surfactant associates with a head-to-tail orientation has been discussed earlier [31,34,35].

**Figure 3:**
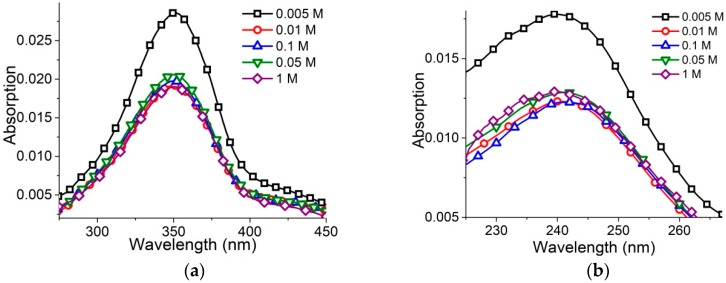
Surfactant absorbance bands (**a**,**b**) at varied [NaCl] (as depicted) and [AzoTAB] = 2 × 10^−6^ M. This surfactant concentration excludes the emergence of micelles even at [NaCl] = 1 M.

Lower surfactant concentration (2 × 10^−6^ M) ensures the absence of micelles even at [NaCl] = 1 M (Figure 3a,b). In this case, surfactant absorption spectra show significant hypochromicity at [NaCl] > 0.005 M without a visible shift of bands.

### 2.2. Effect of Ionic Strength on DNA-AzoTAB Interaction

**Figure 4:**
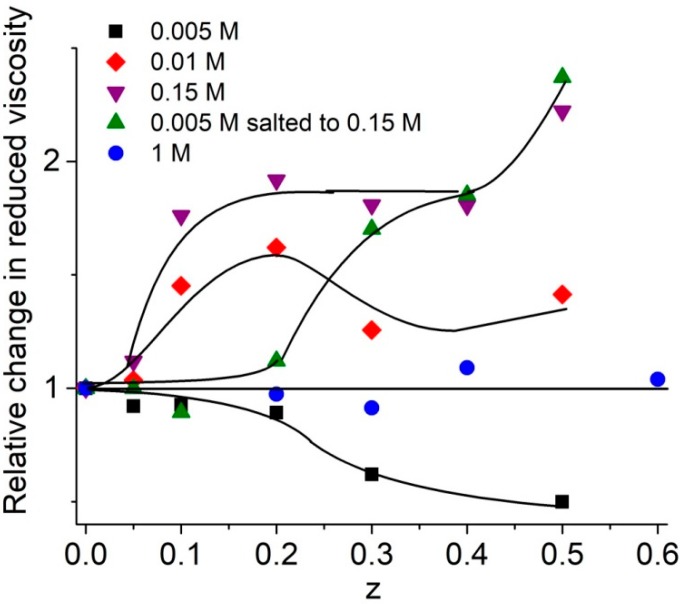
Dependence of relative change in reduced viscosity of DNA solutions on z value at varied [NaCl] (as depicted). [DNA] = 1.5 × 10^−4^ M(P); [DNA] = 2.4 × 10^−4^ M in 1 M NaCl.

In contrast to the low ionic strength solutions, when the addition of surfactant results in a drop of solution viscosity due to the shrinkage of DNA at z < 1, one can see an increase in viscosity in solutions of moderate [NaCl] (0.01–0.5 M) (Figure 4). It should be emphasized that DNA polyelectrolyte swelling is still quite noticeable in these conditions. The binding of positively charged surfactants to DNA should suppress this swelling. Nevertheless, an increase in the solution viscosity indicates the lack of DNA shrinkage. Moreover, the dependence in Figure 4 can testify the formation of macromolecular aggregates in solutions. Intentionally increasing the salt concentration in DNA-surfactant solution prepared in 0.005 M NaCl to 0.15 M results in the same type of dependence as in 0.15 M NaCl (Figure 4). At high [NaCl] > 0.5 M, practically all charges on DNA phosphates are screened, and electrostatic interactions are effectively suppressed. Indeed, at [NaCl] > 0.5 M, surfactant does not affect the DNA molecular volume (the viscosity of DNA solutions does not change). DNA precipitation from the solution with sufficiently high DNA concentrations was observed in this range of [NaCl].

**Figure 5:**
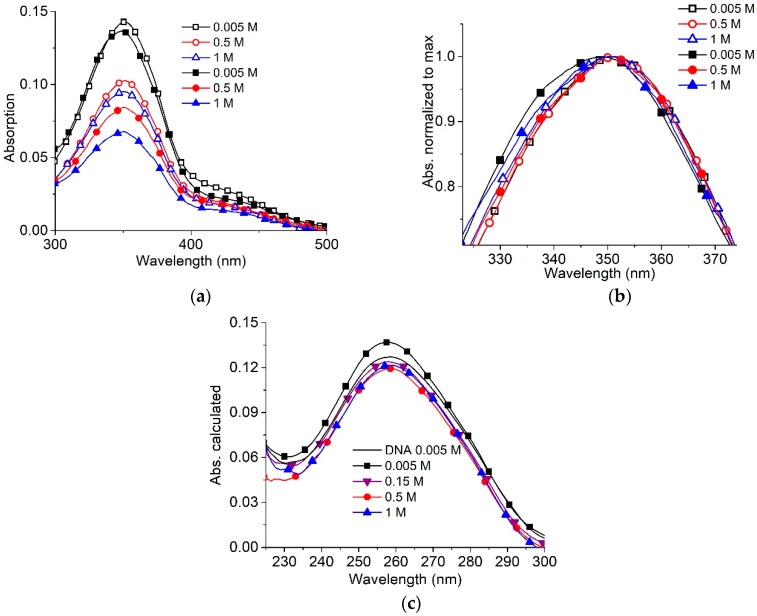
Surfactant absorbance spectra in solutions with DNA at z = 0.5 (filled signs) and without DNA (open signs) at varied [NaCl] (as depicted) (**a**); normalized surfactant absorbance band (**b**); calculated DNA absorbance in complexes (filled signs) and spectrum of free DNA (solid line) (**c**). [AzoTAB] = 2 × 10^−6^ M; [DNA] = 4 × 10^−6^ M (P).

Let us compare surfactant absorption spectra in complexes with DNA at different [NaCl] and z = 0.5 (Figure 5). The low [AzoTAB] prevents the emergence of micelles. Typical changes in surfactant absorption spectra evidence the formation of DNA-surfactant complexes even in 1 M NaCl (Figure 5b). The constant hypsochromic (blue) shift induced by surfactant binding to DNA at varied ionic strengths is observed. Thus, although the viscosity of DNA shows that surfactants do not induce the decrease in volume of DNA coil in 1 M NaCl, spectral data demonstrate the weak binding of surfactant to DNA with the emergence of micelle-like associates on the DNA chain. Calculated DNA spectra in complexes (Figure 5c) demonstrate the unchanged absorption of DNA. Consequently, the DNA bases are not involved in the binding. 

**Figure 6:**
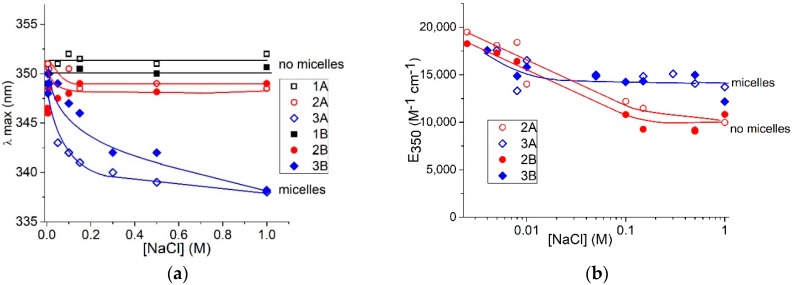
Dependence of hypsochromic shift (**a**) and hypochromic effect (**b**) on [NaCl] for free surfactant (A) and surfactant in complexes with DNA at z = 0.5 (B). [AzoTAB] = 2 × 10^−6^ M (1); 4 × 10^−6^ M (2); 1.8 × 10^−4^ M (3).

The data presented in Figure 6, allow us to compare the surfactant spectral changes induced by varying the salt concentration for free and bound to DNA molecules (at z = 0.5). We have analyzed the surfactant band beyond the DNA absorption region. For surfactant in solutions without DNA (open signs), the hypsochromic shift of band maximum (Figure 6a) and hypochromicity (Figure 6b) at different NaCl concentrations indicate three states of surfactant in solutions, as aforementioned. We can conclude that the first state, predominantly monomeric, exists at low NaCl concentrations. In such an environment, the insufficient screening of surfactant-charged groups presumably prevents the formation of any associates. Salt concentration within the range of 0.01–0.5 M provokes significant hypochromicity in the absorption band without a shift of maximum. That indicates the second state—the emergence of non-micellar associates of surfactant with head-to-tail orientation of molecules. We assume this orientation, since the observed spectral changes for this state differ from those accompanying the formation of micelles (with a predominantly head-to-head orientation). Additionally, in this region of salt concentration, the instability of DNA solutions with a tendency for DNA precipitation can indicate a special state of associates that promotes the formation of DNA-DNA intermolecular contacts. Indeed, due to a small Debye radius ($r_{D}$ < 1 nm at [NaCl] > 0.01 M) the electrostatic repulsion between charged groups ceases, and dimers or other associates with head-to-tail orientation can be formed. The third state of surfactants occurs at [AzoTAB] above CMC. The emergence of micelles manifests itself in hypochromicity and a hypsochromic (blue) shift of the main band maximum (Figure 6) [30]. The red shift of the short-wavelength band is also observed (Figure 2b). The difference between the second and the third states is evident at [AzoTAB] = 4 × 10^−6^ M and lower, when the hypsochromic shift (and, consequently, micelle formation) does not occur at used [NaCl]. The hypochromicity is prominent, indicating the predominance of non-micellar association (second state). Thus, the non-micellar associates, for which hypochromicity without a shift of maximum is typical, exist when [NaCl] provides a sufficiently small Debye radius with the effective shielding of charged groups.

Head-to-head orientation of surfactant in micelles provides the fixed distance between the charged groups and chromophores that contributes to formation of so-called H-dimers [31]. Similar orientation of surfactants is observed in AzoTAB complexes with DNA when the hypsochromic shift of the band maximum occurs (Figure 6a, filled signs). The hypochromicity induced by the increase in [NaCl] for free surfactants and surfactants bound to DNA is similar (Figure 6b). In contrast to the micellar state, the head-to-tail orientation implies variation in relative distance and orientation of chromophores [34]. Thus, the difference in spectral changes for head-to-head and head-to-tail surfactant associates explains the data presented in Figure 2 and Figure 6.

We believe that the head-to-tail orientation of molecules in surfactant associates does not favor the formation of stable DNA-surfactant complexes, preventing the formation of micelle-like surfactant associates on DNA, and provoking DNA aggregation or precipitation. 

### 2.3. The Binding of Di- and Trivalent Metal Ions to DNA in Solutions Containing Surfactants

**Figure 7:**
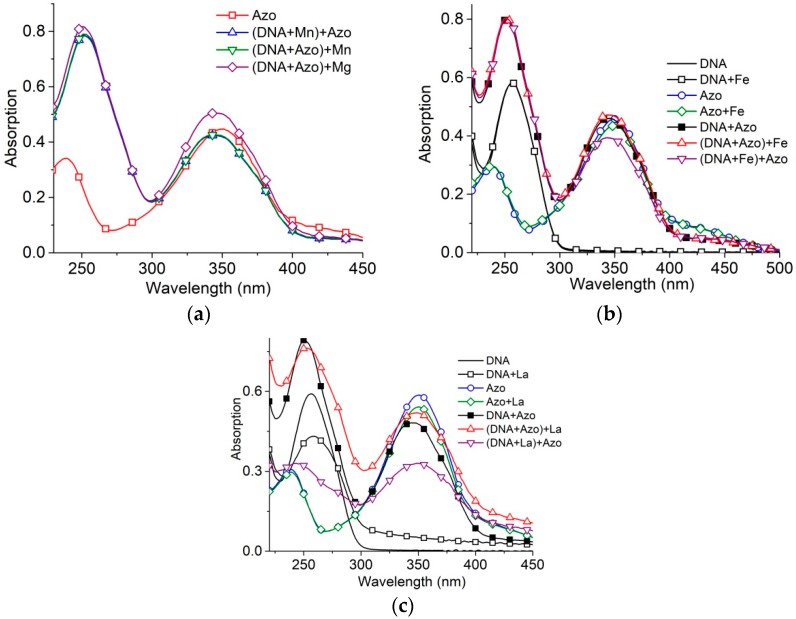
Surfactant absorbance spectra in complexes with DNA in solutions with Mg^2+^ and Mn^2+^ (**a**); Fe^3+^ (**b**); La^3+^ (**c**) in 0.005 M NaCl. Brackets indicate the initial complex. [Mn] = 1 × 10^−5^ M, [Mg] = 2 × 10^−3^ M, [Fe] = 1 × 10^−5^ M, [La] = 1 × 10^−4^ M. [AzoTAB] = 3 × 10^−5^, [DNA] = 9 × 10^−5^ M(P), z = 0.3.

We have considered the influence of two- and trivalent metal ions on the formation of DNA-surfactant complexes. Mg^2+^, Fe^3+^ and La^3+^ are known to interact primarily with DNA phosphates, while Mn^2+^ interacts also with bases in the major groove [36,37]. Experimental data indicate that Mn^2+^ penetrates into a DNA major groove after surfactant binds to DNA, even at a relatively small concentration [Mn^2+^] = 10^−5^ M (hypochromic effect at 260 nm in DNA absorption band with a minor red shift of the band indicate the formation of ion linkage with N7 Guanine). This demonstrates that the formation of the DNA-surfactant complexes do not close the DNA major groove. In the same time, the high concentration of Mg^2+^ (2 × 10^−3^ M) has an insignificant influence on DNA-surfactant complexes (Figure 7a).

The binding of Fe^3+^ to DNA at concentration 10^−5^ M does not prevent significantly further DNA-surfactant interaction (Figure 7b). A similar effect for same concentration was observed for trivalent lanthanum ions. 

Trivalent metal ions at sufficient concentrations can induce DNA condensation (the formation of nanosized particles) [37]. When La^3+^ is present at concentrations causing DNA condensation, 10^−4^ M (Figure 7c), DNA partially escapes from the solution because of phase transition (decrease in the intensity of the DNA absorption band with the emergence of a minor scattering). The decrease in surfactant absorption, because of the addition of surfactant to solutions with condensed DNA, may indicate the binding of a certain fraction of surfactant, which is removed from the solution with DNA-La^3+^ complexes. The addition of La^3+^ to the solution with DNA-surfactant complexes does not cause hypochromicity.

We can conclude that surfactant-DNA complexes are not destroyed after the addition of di- and trivalent metal ions, though the binding of ions with DNA groups in the major groove is possible. Indeed, the association of surfactant tails in micelle-like associates on DNA provides a decrease in the permittivity of medium, stabilizing DNA-surfactant complexes formed by electrostatic attraction of positively charged heads to negatively charged phosphates. Thus, the DNA-surfactants interaction does not affect the major groove of the DNA.

### 2.4. DAPI Luminescence in DNA-Surfactant Solutions

**Figure 8:**
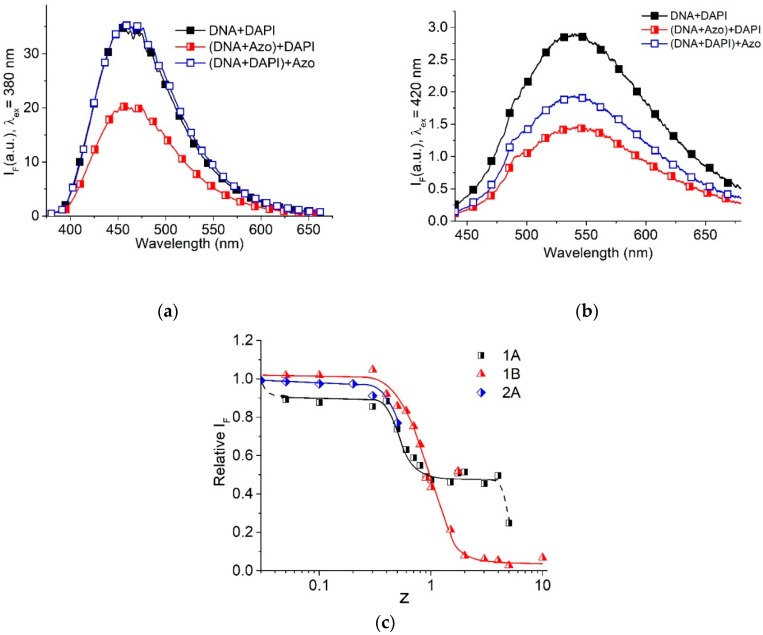
DAPI fluorescence in complexes with DNA before (filled signs) and after (open signs) the addition of surfactant, and after the addition of DAPI to DNA-surfactant complexes (half-filled signs) at r = 0.01 (groove binding), z = 0.3, [DNA] = 4.5 × 10^−5^ M (P), λ_ex_ = 380 nm (**a**); at r = 0.3, z = 0.8, [DNA] = 1.5 × 10^−5^ M (P), λ_ex_ = 420 nm (**b**); dependence of relative intensity of DAPI luminescence on z at 0.005 M NaCl (**c**) at r = 0.3 (1) and 0.03 (2) for λ_ex_ = 380 nm (A) and 420 nm (B), [DNA] = 1.5 × 10^−5^ M (P).

The luminescence of the DAPI dye (4′,6-diamidino-2-phenylindole) in DNA–AzoTAB solutions enabled us to identify the location of surfactant on the DNA helix. DAPI exhibits two types of binding to DNA [38,39] that are unambiguously distinguished with luminescence (Figure 8) [40]. The intense luminescence of DAPI at low r < 0.1 (r is the ratio of DAPI to DNA phosphates molar concentrations) with a maximum at 460 nm and excitation at 340 nm (Figure 8a), indicates the strong A-T-specific DAPI binding to DNA in the minor groove. Once all possible binding sites in the DNA minor groove are occupied, a weaker electrostatic binding of the dye to DNA phosphates takes place. This binding manifests itself well at r = 0.3 as a luminescence with a lower quantum yield, with a maximum at 540 nm and excitation at 420 nm (Figure 8b). The dependences of the relative intensity of DAPI luminescence at 560 nm (excitation at 420 nm) for r = 0.3 (both types of DAPI binding to DNA exist, but luminescence reflects exclusively the DAPI-phosphate binding) and at 460 nm (excitation at 380 nm) for r = 0.03 (minor groove binding) on z at a constant DNA concentration are similar (Figure 8c). The decrease in luminescence, indicating electrostatic binding of DAPI with phosphate groups, demonstrates a competition between DAPI and surfactants for binding sites on DNA. The drop in $I_{420}^{560}$ to zero at z > 2, when DNA compact particles are formed [5], indicates the absence of DNA in the solution. Only free surfactant and free DAPI are spectrally observed in this case. DAPI luminescence at 440 nm $I_{380}^{440}$ decreases weakly at low z and falls sufficiently at z > 0.3 that indicates the hindered groove binding. We believe that at z < 1 the surfactant molecules locate on a long DNA chain in “islets”, and this does not prevent DAPI binding to DNA in the minor groove, although reducing the binding sites. At z > 0.3, the surfactant notably affects the groove binding of DAPI. 

From the analysis of the obtained data, we can conclude that the binding of surfactants to DNA at z > 0.3 prevents the linking of DAPI with phosphates and simultaneously inhibits the binding of DAPI in the minor groove of DNA. Hence, we can assume that the surfactant tails overlap the minor groove of DNA.

### 2.5. DNA Conformation in Surfactant Solutions

**Figure 9:**
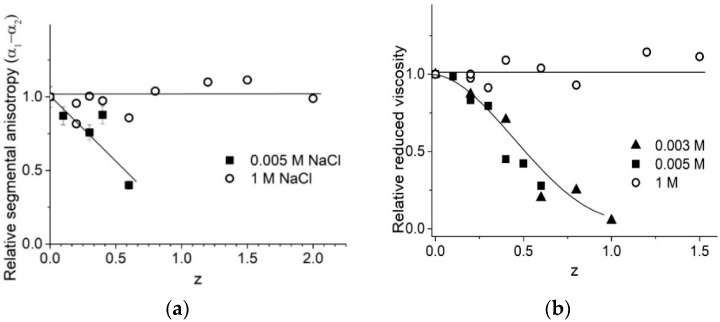
Dependence of relative change in optical anisotropy of DNA statistical segments (**a**) and reduced viscosity of DNA solutions (**b**) on z value at varied [NaCl] (as depicted).

The experimental results demonstrate that we must take into account the possible contribution of surfactant in optical anisotropy of the statistical segment, measured with the flow birefringence technique (Figure 9a). Earlier [5], we believed that the drop in this value was due to a decrease in the DNA stiffness (persistence length) with the shrinkage of the DNA molecular coil being due to the binding of surfactants. Comparing the drops in viscosity (Figure 9b) and in optical anisotropy (Figure 9a), we can conclude according to Formulas (3) and (5) in Section 4, that the observed decrease in (α_1_ − α_2_) caused by the drop of DNA persistence length must induce a more significant decrease in viscosity. Hence, we can conclude that the fall of optical anisotropy is induced by the corresponding orientation of the surfactant tails in complexes with DNA (closed to parallel to the base plane). Noteworthy, the surfactant does not induce any change in DNA optical anisotropy or reduced viscosity of DNA solution at 1 M NaCl.

Finally, we note that the instability of DNA-surfactant solutions at 0.01–0.5 M NaCl and the increase in solution viscosity can be explained by specific orientation of surfactant molecules in associates (head-to-tail orientation), which prevents the formation of micelle-like associates on DNA and provokes DNA aggregation and precipitation. 

## 3. Materials and Methods

### 3.1. Materials

The molecular mass M = 1 × 10^7^ of a high-molecular-weight calf thymus DNA (Sigma-Aldrich, St. Louis, MO, USA) was determined using the value of the DNA intrinsic viscosity [η] (in dL/g) in 0.15 M NaCl, according to [41]:

[η] = 6.9 × 10^−4^ M^0.7^ (1)

DNA was dissolved in Milli-Q ultrapure water; after 2–5 days of storage at 4 °C, salt solution was added to achieve [NaCl] = 0.005 M, with subsequent filtration or centrifugation of the solution. The DNA concentration in stock solution was determined using DNA hydrolysis in 6% HClO_4_ (at 100 °С for 20 min) from the difference in UV absorbance ΔD at 270 and 290 nm [42]. This approach allows us to control the stability of the double helix structure with the value of the molar extinction coefficient by determining the absorption of DNA solutions at 260 nm: E_260_ = 31.1D_260_/[DNA], %. DNA and surfactant solutions at 1 M NaCl and in other NaCl concentrations were prepared by adding an NaCl solution at a higher concentration.

The structure and basic properties of azobenzene trimethylammonium bromide (AzoTAB), M = 476 g/mol, have been described in recent works [5,11,25,30].

We use molar charge ratio z = [AzoTAB]/[DNA phosphates] to describe complexes. The complexes were typically prepared by mixing equal or comparable volumes of solutions.

### 3.2. Viscometry

The relative solution viscosity $\eta_{r}=\frac{\eta }{\eta_{0}}$ (where η and η_0_ are viscosities of the solution and the solvent respectively) was measured at varied velocity gradients g in the range of g = (0.5–2) s^−1^. The η_r_ value at g→0 yields the reduced viscosity of DNA solution $\eta_{\mathrm{red}}=\frac{\eta_{r}-1}{C}$, the values of which at a range of DNA concentrations C allows us to determine DNA intrinsic viscosity [η]:

(2) $$[\eta ]=\underset{C\to 0}{\lim}\frac{{(\eta }_{r}-1)}{C}$$

For DNA with molecular mass M > 2 × 10^6^, the model of swollen statistical coil in a solution is correct, and the Kuhn’s model of polymer chain of hydrodynamic length L consisting of freely jointed segments of length A is suitable, as well as a worm-like chain of persistence length p A = 2p.

DNA is a very rigid macromolecule, but its high charge density leads to non-zero effects of excluded volume—apolyelectrolyte swelling and DNA stiffness determine the state of the coil in the solution:

(3) $$[\eta ]=\frac{\Phi {\langle h^{2}\rangle }^{\frac{3}{2}}}{M}=\Phi \frac{{\left(\mathrm{LA}\right)}^{\frac{3}{2}}}{M}\alpha^{3}$$

In (3), Ф is the Flory parameter, α is the linear swelling coefficient defining the volume effects, including the polyelectrolyte swelling. For the root-mean-square distance between chain ends in real polymer solutions, we have ${\langle h^{2}\rangle }^{\frac{1}{2}}{=(\mathrm{LA})}^{\frac{1}{2}}\alpha$.

In DNA–surfactant solutions, a difficulty arises in maintaining the balance between bound and free surfactant fraction that prevents the accurate calculation of [η].

In our research, we use a viscometric titration. The dependence of reduced viscosity of DNA solutions on the concentration of surfactant (or the z ratio) at a fixed DNA concentration reflects the change in volume of a DNA coil that can be caused by variation in persistence length p and/or in DNA coil polyelectrolyte swelling α.

### 3.3. Flow Birefringence

The birefringence values Δn for DNA solutions were measured in the field of velocity gradient g. The value $\frac{\Delta n/g}{{(\eta }_{r}-{1)\eta }_{0}}$ for g→0 enables us to calculate the optical anisotropy of the DNA statistical segment $\left(\alpha_{1}-\alpha_{2}\right)$:

(4) $$\frac{{\left(\Delta n/g\right)}_{g\to 0}}{(\eta_{r}-1)\eta_{0}}=\frac{4\pi }{45{\mathrm{kTn}}_{s}}\frac{{\left(n_{s}^{2}-1\right)}^{2}}{n_{s}}\left(\alpha_{1}-\alpha_{2}\right)$$

In (4), n_s_ is the solvent refractive index, k is the Boltzmann constant, T is the absolute temperature, $\left(\alpha_{1}-\alpha_{2}\right)$ is proportional to the average optical anisotropy of a DNA base pair Δβ and the number of base pairs in statistical segment S (DNA rigidity):

(5) $$(\alpha_{1}-\alpha_{2})=S\Delta \beta$$

### 3.4. Spectral Methods

UV/Vis absorption spectra were recorded using SF-56 spectrophotometer (LOMO, Russia). The luminescence spectra of DAPI and EB were measured using the Hitachi-850 spectrometer in a 1 cm quartz cell, after the solutions were stored for at least 1 h at room temperature. Luminescence excitation and emission spectra were corrected for spectral sensitivity of the instrument.

## 4. Conclusions

The molar charge ratio of surfactant and DNA concentrations and the solution ionic strength determine the formation of three different systems: A solution with DNA-surfactant complexes, DNA precipitation with the emergence of large flakes and a disperse system with nanosized particles. NaCl concentration was demonstrated to determine the surfactant-surfactant interaction in solutions without DNA: The prevalence of non-micellar associates (with head-to-tail orientation) was found at [NaCl] > 0.01 M, whereas such structures are not typical at low ionic strength. For the first time, in sufficiently concentrated AzoTAB solutions at 0.01 M < [NaCl] < 0.5 M, we found DNA aggregation to be enhanced, occurring at surfactant/DNA ratios < 1. Low [NaCl] < 0.01 M promotes the formation of a complex with emergence micelle-like surfactant associates on DNA, while [NaCl] = 1 M prevents the electrostatic attraction of surfactants to the phosphate groups of DNA for the formation of complexes. It was observed that surfactant-DNA complexes are not destroyed by the addition of di- and trivalent metal ions into the solution; however, the binding of metal ions in the DNA major groove is possible after DNA-surfactant binding. To determine the location of surfactant molecules on DNA, the luminescence of the DAPI dye was studied upon its addition to solutions with DNA-surfactant complexes: The association of surfactants on DNA prevents DAPI binding to phosphates and inhibits the dye binding in the DNA minor groove. Hence, we assume that the surfactant tails overlap the minor groove of DNA. Nevertheless, the luminescence of the minor groove-bound DAPI does not change after surfactant addition into solution with DNA-DAPI complexes. The change in the volume of a DNA molecular coil in complexes with surfactants (DNA packaging at [NaCl] < 0.01, DNA aggregation at [NaCl] > 0.01 and the invariability of DNA volume at [NaCl] > 0.5) was analyzed by the viscometry method. Decrease in DNA optical anisotropy accompanies the drop in viscosity. This is due to the fall in DNA persistence length and/or the contribution of the surfactant to the measured optical anisotropy of the DNA statistical segment. 

In conclusion, it should be emphasized that our research could be of interest for further developments related to the use of such surfactants. An example of a DNA response to the presence of various surfactant associates in the solution (with head-to-head or head-to-tail orientation) shows that we can prevent polymer aggregation in solution by controlling salt concentration. Of particular interest is the study of cis-trans isomerization of such surfactants in solutions of different ionic strength. Such studies were performed earlier, but taking into account the obtained data, the role of different surfactant associates in the reversible DNA condensation can be more thoroughly studied.
